# Resilient Response of Cement-Treated Coarse Post-Glacial Soil to Cyclic Load

**DOI:** 10.3390/ma14216495

**Published:** 2021-10-29

**Authors:** Katarzyna Zabielska-Adamska, Mariola Wasil, Patryk Dobrzycki

**Affiliations:** Department of Geotechnics and Structural Mechanics, Faculty of Civil Engineering and Environmental Sciences, Bialystok University of Technology, ul. Wiejska 45E, 15-351 Bialystok, Poland; m.wasil@pb.edu.pl

**Keywords:** resilient modulus, cyclic loading, compacted soil, cement stabilisation, bound base and subbase

## Abstract

Stabilisation with cement is an effective way to increase the stiffness of base and subbase layers and to improve the rutting of subgrade. The aim of the study is to investigate the effect of different percentages of cement additives (1.5%, 3.0%, 4.5% and 6.0%) on the resilient modulus of coarse-grained soil used on road foundations. The influence of the compaction method, the standard Proctor and the modified Proctor, as well as the sample curing time is analysed. The cement addition significantly increases the resilient modulus and reduces the resilient axial strain. Extending the curing time from 7 to 28 days also improves the resilient modulus. The change in the compaction energy from standard to modified does not increase the resilient modulus of the stabilised gravelly sand due to its compaction characteristics. The test results of the resilient modulus of the gravelly sand stabilised with cement indicate the possibility of using it as a material for the road base and subbase due to meeting the AASHTO requirements. However, the non-stabilised gravelly sand does not meet the above requirements. It has been sheared during cyclic tests at the first load sequence, regardless of the compaction method.

## 1. Introduction

The road surface is a layered composition made of processed and compacted materials of various thicknesses. It creates a structure that transfers the loads from vehicles in a specific time and enables comfortable driving. In general, we can distinguish pavements (flexible bituminous, rigid concrete and composite pavements) where asphalt or cement-bound material is used beneath the asphaltic concrete surface. Flexible pavements also include semi-rigid pavements, where the base is built from bound material. Pavement foundation can be defined as one or more layers of compacted unbound or asphalt or hydraulically bound granular material placed over the subgrade soil. The soils and granular materials in the pavements are exposed to a large number of loads at stress levels much below their shear strength. Under a single load on a moving wheel, the pavement reacts in a significantly elastic manner. However, with repeated loading, irreversible plastic and viscous stresses can accumulate [1], and the thickness of the pavement layers is of significant importance in the behaviour of flexible pavements.

The effect of repetitive loads on the pavement structure was first described by Hveem [2]. Hveem introduced the parameter “resilience”—compression and rebound under passing loads that were measured by means of a new device—resiliometer—where the deformation of a sample in repeated loading is measured as a volumetric displacement. The resilience of the underlying materials was indicated as one of the three main design parameters, in addition to expansion and plastic deformation, describing the actual behaviour of the pavement. The resiliometer was a modification of the stabilometer (still used today), differing in repetitive load. Seed et al. [3,4] defined the resilient response of granular material as the modulus of resilience (next called modulus of resilient deformation and resilient modulus), that is, the value of repeated applied deviator stress in triaxial compression divided by the recoverable (resilient) axial strain:

(1) $$M_{r}=\frac{\sigma_{d}}{\varepsilon_{r}}=\frac{\sigma_{1}-\sigma_{3}}{\varepsilon_{r}}$$

which is equivalent to the elastic Young’s modulus. However, under cyclic loading, the modulus of elasticity can be replaced with the resilient modulus to account for the non-linearity and stress relationship during cyclic loading. The resilient modulus was tested in developed a triaxial apparatus with the possibility of a repeated load of the sample.

The currently used methods of mechanistic design for pavement and pavement layers require the resilient modulus of unbonded pavement layers to determine layer thickness and the overall system response to traffic loads in the case of flexible pavements. The American Association of State Highway and Transportation Officials (AASHTO) adopted this parameter in 1986, issuing guidelines “AASHTO Guide for Design of Pavement Structures” [5], and also relying on it in subsequent editions. The method of determining the parameter is given in the standard AASHTO T 307-99 [6], and the formula for calculating the resilient modulus corresponds to Equation (1):

(2) $$M_{r}=\frac{\sigma_{cyclic}}{\varepsilon_{r}}$$

where $\sigma_{cyclic}\;$ is the amplitude of cyclic applied axial stress and $\varepsilon_{r}$ is the relative resilient (recovered) axial strain.

**Figure 1:**
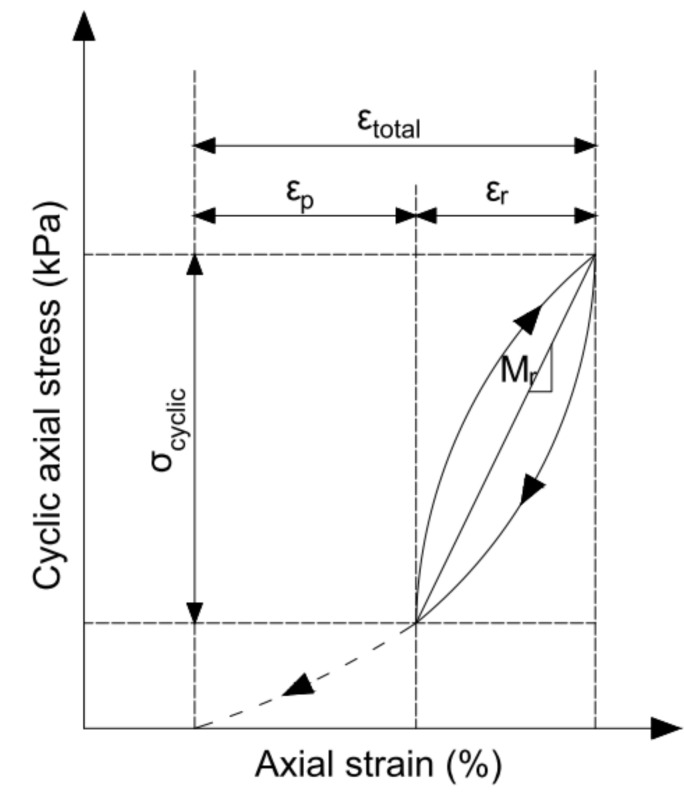
The cyclic load response in a single cycle in the resilient modulus test, where $\varepsilon_{p}$ is the permanent relative strain and $\varepsilon_{r}$ is the resilient relative strain.

Figure 1 shows the interpretation of the resilient modulus in a repeated-load triaxial test.

Until now, many researchers have studied the influence of several factors on the resilient modulus of different soil types. In the laboratory, the resilient modulus of unbound materials has been most often assessed by applying a triaxial apparatus, but other methods, including the simple shear test, torsional resonant column testing, hollow cylinders and true triaxial tests, can be used, too. The influence of the deviator stress and confining pressure, the load duration and frequency, the number of load cycles, material graining and density, and the degree of moisture content was described in detail by Brown [1] and Lekarp et al. [7,8]. They also cited various mathematical models from the literature for predicting the resilient response of materials under repeated loading, with an indication of their advantages and limitations. In addition, they showed permanent strain modelling with respect to the number of load applications and stress conditions.

Among the numerous works on the non-linear behaviour of cyclically loaded soils and granular materials, some publications have to be mentioned. Many authors pay attention to the fact that the main factor influencing the resilient modulus of untreated granular soils is the stress level, inter alia [4,9,10,11,12,13], both confining pressure and applied axial stress. This phenomenon was also stated in the case of cohesive soils [14,15,16,17]. In all cases, the resilient modulus increased significantly with the confining pressure and marginally with the repeated axial stress.

The resilient modulus increases with a rise in the number of cycles, but it decreases with growing frequency [18]. Tanimoto and Nishi [14] noted that the resilient strains of silty clay after large numbers of repetitions achieved a constant value after changing the soil structure. Tang et al. [13] estimated the repetition quantity for approximately 100 cycles. The variability of the accumulated plastic strain was greater with a greater number of load cycles, higher amplitude of dynamic stresses and lower confining pressure.

Hicks and Monismith [9] found for coarse soil that the sample saturation during the tests had an insignificant impact on the resilient modulus. At a given stress level, the modulus was higher in the dry and saturated condition; in the case of an average value of the degree of saturation, the modulus was lower. A greater influence of saturation was stated for the resilient Poisson’s ratio. The increase in the moisture content during the compaction of fine-grained soil reduces the value of the resilient modulus [19]. Analysis of fine-grained soil with low plasticity [20] showed that the resilient modulus increases with the increase in matric suction and relative compaction. Thus, a lower moisture content and high relative compaction favourably affect the stiffness of cohesive soils, which is commonly known.

The test conditions are also particularly important when testing the deformation of saturated samples [1]. The resilient modulus determined from the internal and external deflection measurements may differ significantly [21]. The difference is less than 50% for unbound materials and subgrade soils, but it is higher for stiff, stabilised materials.

The resilient response of bounded soils is rather poorly presented in the literature. The effect of lime on the stiffness of fine-grained soils is described more often. The resilient response of fine-grained soils treated with lime is visible immediately after sample preparation for uncured soils [19]. The 5% lime addition provides a higher resistance to repeated loads of soils with high moisture contents and soils subjected to freeze-thaw cycling. Bhuvaneshwari et al. [22] evaluated the influence of lime addition and time of curing on resilient modulus values. They observed $M_{r}\;$ increasing with a percentage quantity of lime (0–8%). In the case of lime addition in percentages of 4%, 6% and 8%, the hardening time effect is visible. Thompson [23] described lime-reactive soils that show a significant increase in strength over time as reactive and those showing limited pozzolanic reactivity as non-reactive. Yuan et al. [24] assessed the same lime and cement additions as improving agents of specific fine-grained soils—red clay. They found that the dynamic resilient modulus of red clay stabilised with cement is slightly higher in comparison to that with lime. The laboratory tests were conducted on specimens compacted at the optimum water content ±3%. Ismail et al. [25] tested crushed granite aggregates with the finest content of about 8%, stabilised by various cement additives from 0% to 6%. Specimens were tested by the indirect tension method for the resilient modulus of bituminous mixtures, so test results are difficult to compare. The $M_{r}\;$ value increases in proportion to the percentage of cement added. For samples cured for 7, 28 and 60 days, the fastest increase in $M_{r}\;$ was observed between 7 and 28 days, whereupon a further increase was observed. Hanifa et al. [26] analysed two cohesive soils with 6–8% of cement and one with 10% of cement, compacted at *w*_opt_ ± 2%. The resilient modulus increased with curing time, and the greatest values were obtained at optimum water contents.

Cement stabilisation of granular layers is an effective technique of increasing the stiffness of base and subbase layers. In addition, cementitious bases can improve the fatigue properties of asphalt layers and subgrade rutting in the short and the long term. However, it can provide additional stresses, such as shrinkage and fatigue of the stabilised layers [27]. Portland cement can be used for a wide range of soils, from low to moderately high plasticity, to modify or improve soils, but the best results are obtained for well-graded materials. Based on the literature [28], the plasticity index of cement-stabilised soil should be less than 30 for sand soils and less than 20 for fine-grained soils (for which the liquid limit should be less than 40). This condition is connected with the proper mixing of the stabiliser. For granular materials, at least 45% of the soil should pass through a 12.5 mm sieve. According to AASHTO recommendations [29], the amount of cement depends on the required final soil properties, and the minimum cement content varies from 3% to 16%, relating to the kind of soil, where the latter value seems to be over-stabilising. It should be noted that the European standard EN 14227-15 [30] does not limit the soil graining for the cement-bound soil incorporated into the road structure. The cement content is also not limited, and the properties of the stabilised soil are an indicator of correct application. It is assumed that the minimum amounts of cement, depending on the maximum grain size of the soil/aggregate, are 3–5%.

The aim of the study is to investigate the influence of various percentages of cement additives (1.5%, 3.0%, 4.5% and 6.0%) on the resilient response of coarse post-glacial soil used for the construction of road base and subbase. Cement additives were selected as the minimum amounts that can improve the resilient properties of the tested soil and not deteriorate shrinkage and fatigue after material incorporation into the road structure. The effect of the compaction method (the standard and modified Proctor methods) and the curing time of stabilised samples will be determined. The relative resilient axial strain will be shown for all load cycles, which explains the values of the resilient modulus.

## 2. Materials and Methods

### 2.1. Materials

**Figure 2:**
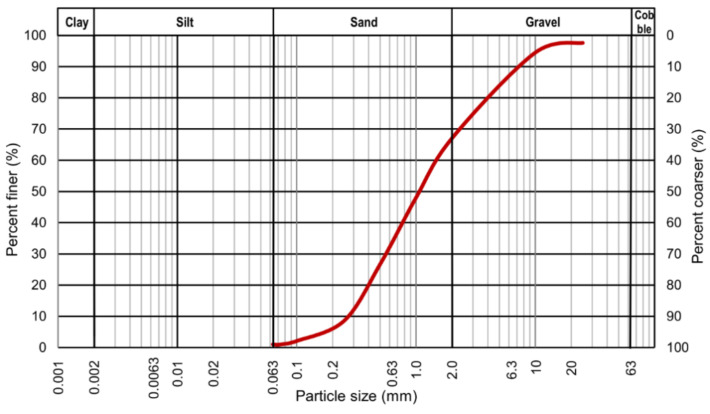
Grain size distribution curve of the tested granular soil.

Laboratory tests of the resilient modulus were conducted on granular soil and soil–cement mixtures—hydraulically bound mixtures (HBM). The cement that was added to the mixture was commercially available Portland cement 42.5R. Figure 2 shows the grain size distribution curve of the tested soil, which was obtained from the sieve analysis performed according to the EN 933-1 standard [31].

As observed (see Figure 2), the assessed soil is a coarse soil. The primary fraction is sand, and the secondary is gravel. According to the EN ISO 14688-1 standard [32], the presented soil is gravelly sand (grSa). Based on the grain size distribution curve, the coefficient of uniformity, *C*_U_, and the coefficient of curvature, *C*_C_, were calculated. The values of *C*_U_ and *C*_C_ were as follows: 5.45 and 0.87, respectively. This indicates that the assessed coarse soil is poorly graded [33]. The tested soil meets the requirements of EN 13242 [34] and ASTM D1241 [35] for subbase or base materials (gradation D) with a lower percentage of fine fractions, which is required in the frost area.

**Figure 3:**
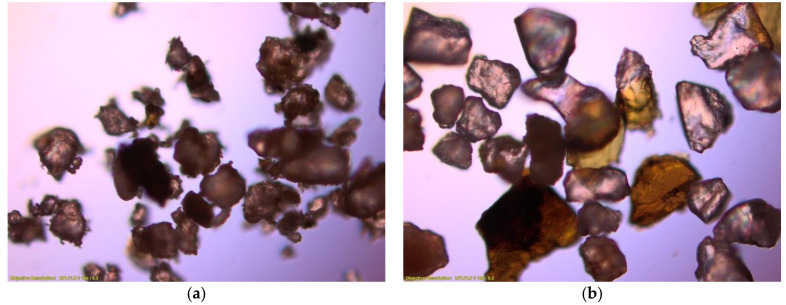
Optical micrographs (×100) of the dry soil (**a**) and the soil washed with water (**b**).

Gravelly sand, as a glaciofluvial soil (Pleistocene), is characterised by a large variation of the relief surface, which is associated with high dynamics of the sedimentary environment and the diversity of the mineral composition in the case of post-glacial soils. Apart from well-rounded quartz crumbles, there are also angular grains, with a substantial addition of lytic particles and feldspars [36]. Optical fluorescence microscope images were taken to check the composition of the tested material. Figure 3a shows the natural dried material, and Figure 3b shows the material washed with water and then dried.

Comparing the images, it can be observed that the smaller grains adhere to the larger grains, despite the absence of the finest particles—clay and silt. Thanks to the image in Figure 3b, it is possible to assess the shape of the grains, their texture and, above all, their mineral composition, as described in the case of glaciofluvial soil.

**Figure 4:**
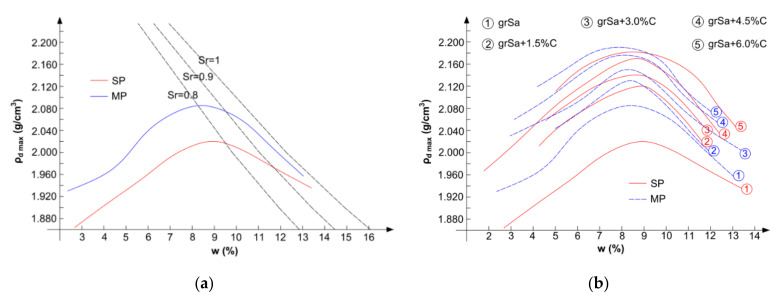
Compaction curves obtained by the standard Proctor (SP) and modified Proctor methods: (**a**) gravelly sand and (**b**) gravelly sand and its mixtures with cement addition (C).

The optimum moisture content, *w*_opt_, and the maximum dry density, *ρ*_d max_, of the tested materials were established due to two compaction methods. The standard and modified Proctor compaction tests were conducted following the EN 13286-2 standard [37]. The compaction curves of coarse soil with saturation lines are presented in Figure 4a. The compaction curves of gravelly sand and its mixtures with cement are shown in Figure 4b.

As can be seen in Figure 4a, the value of *ρ*_d max_ obtained from the standard Proctor compaction is lower than the one obtained by the modified method. In the case of material compacted with higher compaction energy, the degree of saturation, *S*_r_, is higher than that for soil compacted with lower compaction energy.

In the case of both compaction methods, with a higher amount of cement in the mixture, the *w*_opt_ value decreased, while the *ρ*_d max_ value increased. In general, by the standard Proctor method, the obtained values of the optimum moisture content were higher, and values of the maximum dry density were lower. This was confirmed by the results of the authors’ research and other results from the literature [38,39]. However, it should be noted that the differences in the compaction parameters of the samples stabilised with cement were small for both compaction methods (see Figure 4b).

The compaction parameters, *w*_opt_ and *ρ*_d max_; the initial void ratio, *e*; and the specific dry density, *ρ*_s_, of tested materials are presented in Table 1.

It can be concluded that for both compaction methods, the value of *e* decreases with the increase in the cement addition in the mixture. In general, the specific dry density of the materials increases with the higher amount of cement in the mixture.

### 2.2. Methods

Laboratory tests of the resilient modulus of coarse soil and coarse soil–cement mixtures were performed in the cyclic triaxial test apparatus. In the device, the confining pressure and axial load were applied pneumatically. The machine applied repeated cycles of the haversine-shaped load pulse, where the load pulse lasted 0.1 s and the rest period 0.9 s.

Tests were conducted on compacted samples of gravelly sand and gravelly sand–cement mixtures with variable percentages of cement additions. The amounts of the cement in the mixture were 1.5%, 3.0%, 4.5% and 6.0%. The percentage represents the dry mass of the cement per dry mass of coarse soil in a tested sample. First, dried soil was mixed with the cement. The next step was to add an adequate amount of water to obtain a moisture content corresponding to the *w*_opt_ for each material. The cylindrical samples were prepared in a bipartite mould by compaction of the material into three layers. The diameters, *D*, of the samples were about 70 mm, and the heights, *H*, were two times greater, about 140 mm. The amounts of the soil and soil–cement mixtures taken to prepare samples were adequate to obtain the *ρ*_d max_ value derived from the standard and modified Proctor methods (see Table 1). The samples were tested directly after compaction (unstabilised hydraulically) and after 7 and 28 days of curing at constant temperature and humidity, wrapped in foil to avoid drying.

**Figure 5:**
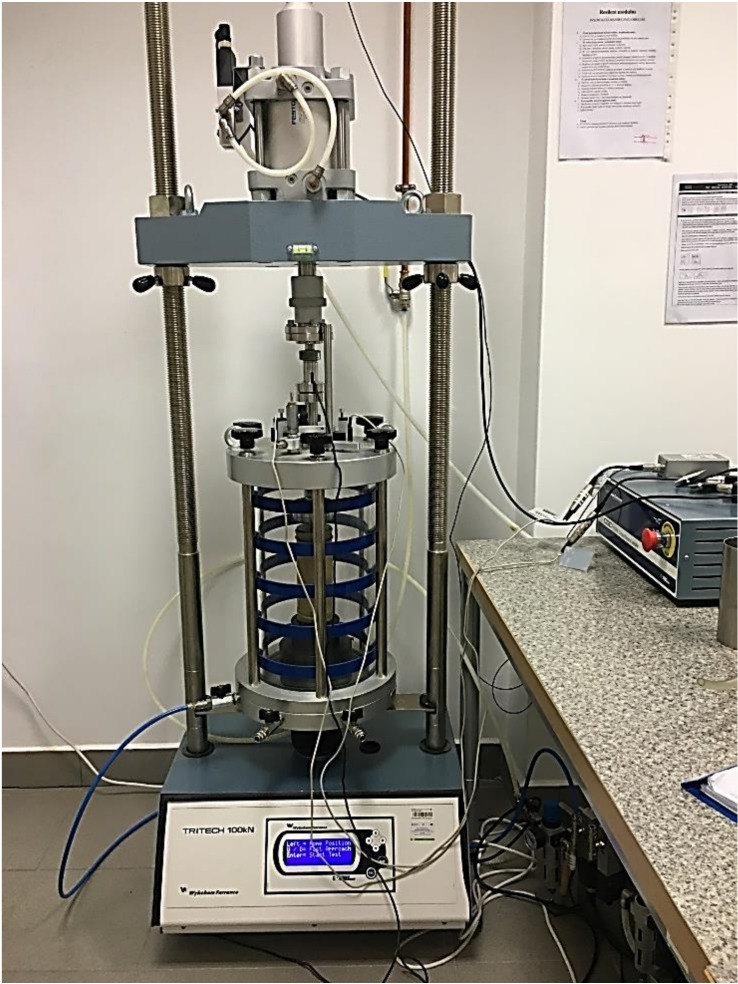
View of the cyclic triaxial apparatus with the sample in a cell before starting the test.

The prepared samples were secured on both ends by filter paper. On the base pedestal of a triaxial cell, a sample was placed with a porous disc on the bottom and the top. A rubber membrane was placed over the sample and sealed on the bottom by O-ring sealing. Next, the top cap was seated and sealed by the O-ring. The main body of the cell was assembled, and the load piston was tightened with a screw to an actuator. The piston was slowly moved down to contact the top cap. The changes in the sample height during the tests were measured by external LVDT displacement transducers. The test settings and results obtained were controlled and saved by a computer programme. The assembly of the machine with the sample is presented in Figure 5.

Samples were subjected to cyclic loading tests to determine the resilient modulus $M_{r}$ according to the AASHTO T307 standard [6]. Table 2 presents the sequence information for subgrade material. Sequence 0 is the conditioning of the sample. In the next 15 sequences, the confining pressures ranged from 20.7 to 137.9 kPa and the maximum axial stresses ranged from 20.7 to 275.8 kPa. The resilient modulus $M_{r}$ for sequences from 1 to 15 was calculated as the average value from the past five cycles of each load sequence.

## 3. Results and Discussion

**Figure 6:**
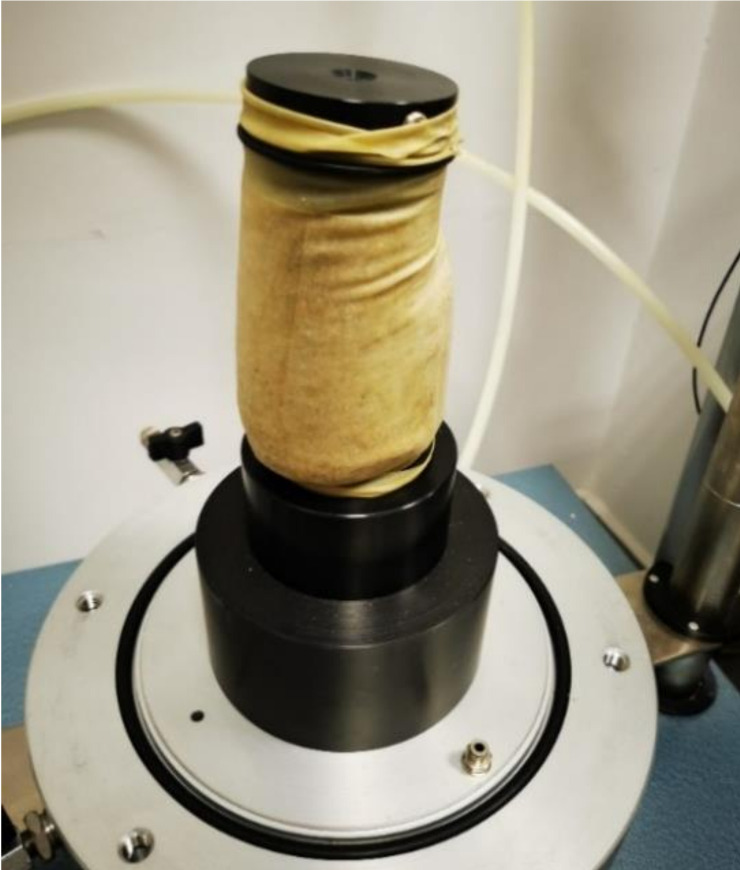
View of the damaged gravelly sand sample during test sequence 1.

Figure 6 presents a gravelly sand sample subjected to the resilient modulus test. The sample was damaged by shearing after just the first test sequence. Several tests did not allow determining the $M_{r}$ value of gravelly sand without the addition of cement. The destruction of an unstabilised sample in a cyclic test proves its non-resistance to cyclical interactions and the correct decision to stabilise the material with cement in the case of embedding the tested material in road foundations.

**Figure 7:**
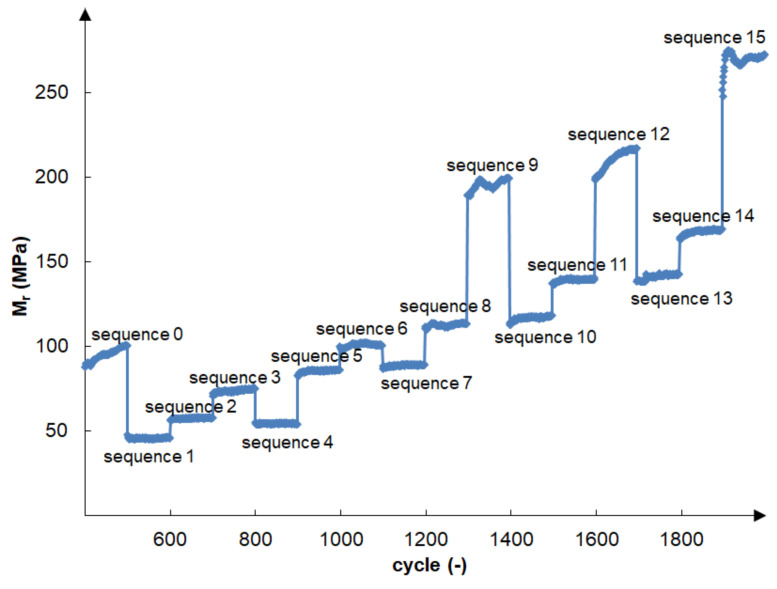
Variation in the resilient modulus in all test cycles (see Table 2) for sample (grSa+3.0%C) compacted by the MP method and cured for 7 days.

The cement-stabilised gravelly sand did not deteriorate during cyclic loading in accordance with the AASHTO T 307-99 recommendation [6], even with a minimum 1.5% cement addition. The effect of variation of confining pressure, $\sigma_{3},$ and the maximum applied axial stress on resilient modulus, $M_{r},$ in the successive test cycles in accordance with Table 2, for an exemplary bound sample (grSa+3.0%C) is shown in Figure 7. The presented sample was compacted by the MP method and cured for 7 days. $M_{r}$ initially increased rapidly and became almost constant after 100 cycles of load applications. The initial increase in $M_{r}$ can be explained by cyclic sample compaction under increasing maximal axial stress. The greatest increase in $M_{r}$ could be observed during sequence 0, and sequences 12 and 15 when there was the greatest increase in axial stress. The highest values of $M_{r}$ were obtained under the load of sequence 15, where the confining stress and the maximum applied axial stress were the highest: 137.9 kPa and 275.8 kPa, respectively. The lowest values of $M_{r}$ were obtained during sequence 1 when the load was the lowest, so the confining stress and the maximum applied axial stress were 20.7 kPa.

**Figure 8:**
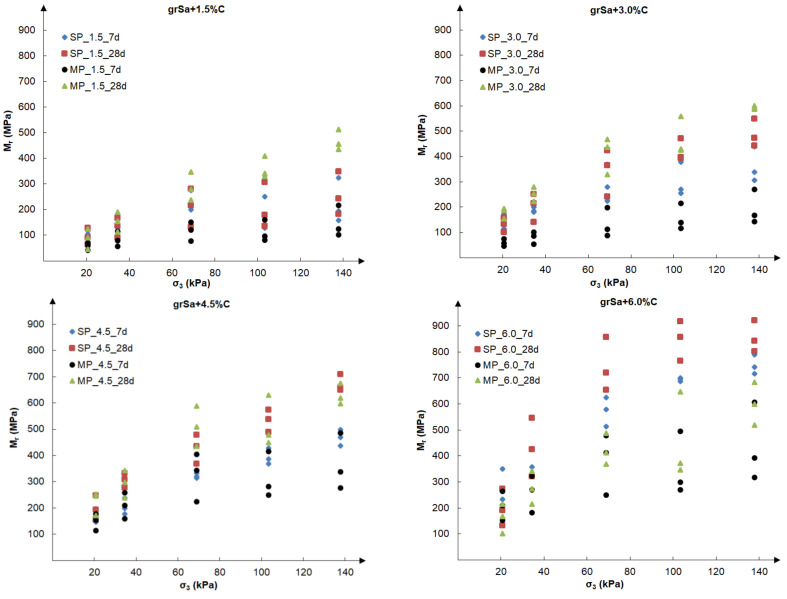
The results of final resilient modulus vs. confining pressure obtained for samples compacted by the SP and MP methods after 7 and 28 days of curing for the various cement additives.

Subsequent analysis was performed to evaluate the influence of the test conditions and circumstances for the preparation of samples, i.e., compaction method, cement addition and time of curing, on the values of the resilient modulus. Figure 8 presents the final resilient modulus, calculated as the average value from the past five cycles of each load sequence, as a function of five different confining pressures, taken according to Table 2, i.e., 20.7, 34.5, 68.9, 103.4 and 137.9 kPa. For each examined sample, three different load sequences were performed at the same confining pressure. The test results are shown separately for each cement additive: 1.5%, 3.0%, 4.5% and 6.0%.

The resilient modulus increased with rising confining pressure and with the same confining pressure, with rising maximum axial load, which can be observed in Figure 7 and Figure 8. Specimens stabilised with 1.5–4.5% of cement, tested at the same confining pressure (see Figure 8), gained the highest values of $M_{r}$ for the modified compaction. This was especially visible at greater values of the confining pressure. The exception was the 6% cement addition, for which the highest $M_{r}$ values were obtained at the standard compaction.

**Figure 9:**
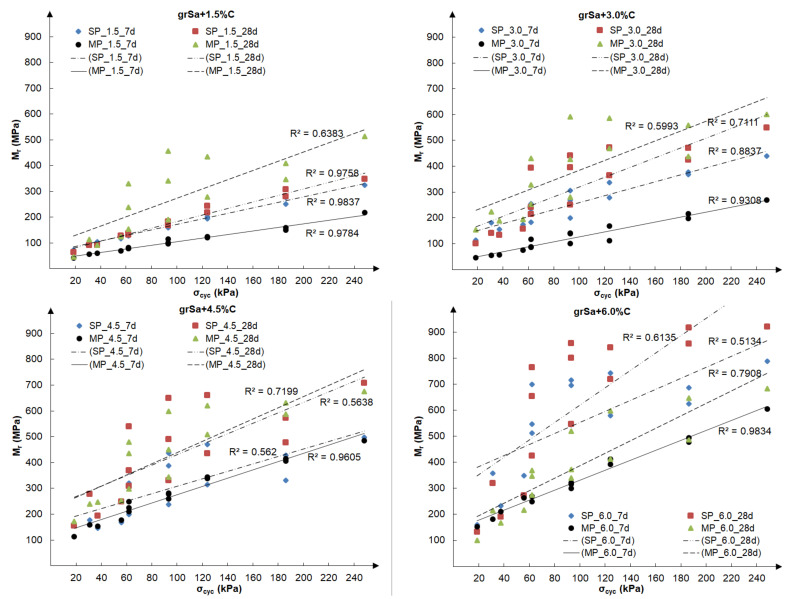
The results of final resilient modulus vs. cyclic applied axial stress obtained for samples compacted by the SP and MP methods after 7 and 28 days of curing for the various cement additives.

Figure 9 shows the relationship between the final resilient modulus and cyclic applied axial stress, calculated for all tested samples.

Based on Figure 8 and Figure 9, it can be concluded that the addition of cement has a considerable influence on the increase in the value of the resilient modulus. Increasing the cement addition by 1.5% resulted in an approximately 50% increase in the resilient modulus value in most cases assessed for the same sequence. In the case of the tested soil, the method of sample compaction was less important, and the results obtained for both compaction methods, standard Proctor and modified Proctor, were comparable, although generally slightly higher for samples compacted by the means of the modified method. However, it should be noted that in the case of samples stabilised with the same cement additive, the compaction curves correspond to each other (see Figure 4b), and the obtained void ratios at optimum water content are similar (see Table 1). The influence of the curing time is visible in the case of samples compacted by the modified Proctor method. The samples cured for 28 days had a higher $M_{r}$ than those cured for 7 days. Samples stabilised with 1.5–6.0% cement addition reached the resilient modulus values within 42–789 MPa after 7 days of curing and 46–921 MPa after 28 days. The samples compacted by the standard Proctor method showed this only with higher cement additions, i.e., 4.5% and 6%. At 6% cement addition, the greatest dispersion of the obtained $M_{r}$ results in dependence on confining pressure is observed, which can be seen in Figure 8. In the case of the $M_{r}\left(\sigma_{cyclic}\right)$ relationship (see Figure 9), the lowest coefficients of determination, $R^{2}$, but explaining over 50% of variables, were found for samples with different cement additives cured for 28 days. In both cases, it may be caused by the drying out of the specimens during curing.

The obtained test results confirmed the beneficial effect of the amount of cement and the hardening time of the stabilised samples. In the case of cement-stabilised cohesive soils, an increase in the $M_{r}$ value was also observed after increasing the cement addition and extending the curing time [25,26], as in the case of lime-stabilised cohesive soils [22].

**Figure 10:**
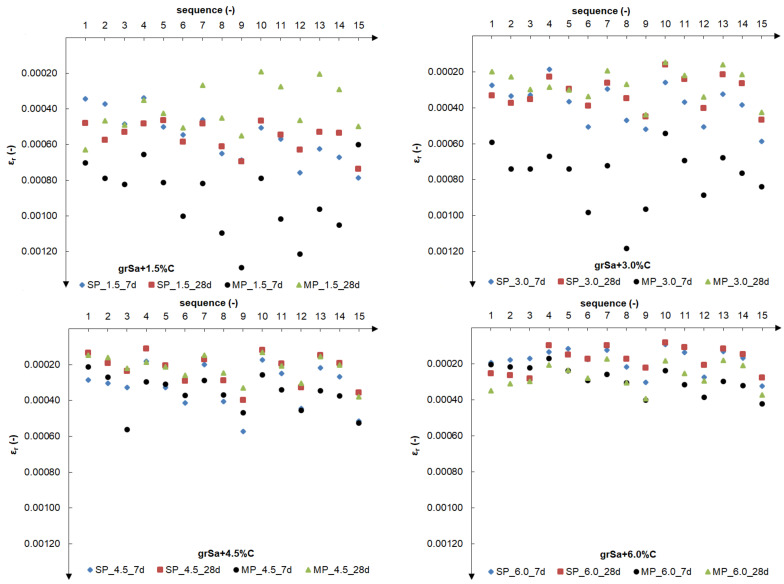
The final values of the relative resilient axial strain in particular 1–15 load sequences for samples compacted by the SP and MP methods after 7 and 28 days of curing for the various cement additives.

Figure 10 shows changes in the final values of the relative resilient axial strain in particular load sequences.

The results were obtained as average values from the past 5 cycles from 100 cycles of each load sequence (1–15) according to Table 2.

For cement additives (1.5%, 3.0%, 4.5% and 6.0%), the influence of cyclic test conditions was visible (see Figure 10). In the next three sequences, a constant confining pressure was assumed, and the maximum applied axial stress was increased successively. The graphs clearly show the results arranged in a series of three points. The greater the cement addition, the more this phenomenon is visible. With the increase in cement addition, increasingly smaller relative axial deformations were observed, i.e., with the addition of 1.5%, 0.034–0.121% relative axial deformation; 3.0%, 0.020–0.112%; 4.5%, 0.015–0.057%; and 6.0%, 0.008–0.042%. Thus, the addition of 1.5% cement caused a decrease in the value of relative axial strain up to several dozen percentage points. This is the main reason for the increase in the value of the resilient modulus.

In future research, we plan to perform tests with a greater number of repetitions of cyclic loads in order to determine the accumulated permanent strain and to classify the tested geomaterials in terms of shakedown theory [40,41,42].

## 4. Conclusions

Based on the test results of compacted gravelly sand in a natural state and stabilised with cement, the following conclusions are obtained:

(1) The results of research on the resilient modulus of gravelly sand stabilised with cement indicated the possibility of using it as a material for road engineering applications. The tested soil, compacted to obtain optimum compaction parameters, can meet the AASHTO requirements [5] formulated for granular foundations as 207 MPa for both flexible and rigid road pavement structures, especially in the case of greater cement addition.
(2) Tested non-stabilised gravelly sand as a poorly graded material does not meet the above requirements. The tested samples compacted at the optimum water content to the maximum dry density were sheared during cyclic tests during load sequence 1 regardless of the standard Proctor or the modified Proctor compaction method.
(3) Cement addition significantly increases the obtained values of the resilient modulus and reduces the relative resilient axial strain. Extending the curing time of the sample from 7 to 28 days also affects the value of the resilient modulus, increasing it. Another increase in the addition of 1.5% of cement reduces the value of the relative axial deformation even by several dozen percentage points, which is the main explanation of the increase in the value of the resilient modulus.
(4) In the case of the tested gravelly sand, increasing the compaction energy from standard to modified does not, in principle, increase the resilient modulus value, which is caused by the specific compaction characteristics of stabilised soil. For samples stabilised with the same cement additive, the compaction curves correspond to each other. The void ratios obtained at optimum water content are similar.
(5) Test conditions, the confining pressure and the maximum applied axial stress affect the obtained resilient modulus values, which was also indicated in previous publications. The resilient modulus increases with an increase in confining pressure and applied axial stress 3.9–11.2 times (average 5.4 times) for various cement additives, compaction methods and sample curing times.

## Figures and Tables

**Table 1:** Geotechnical properties of tested materials.

Material	Compaction Method						*ρ*_s_ (g/cm^3^)
	Standard Proctor			Modified Proctor			
	*w*_opt_ (%)	*ρ*_d max_ (g/cm^3^)	*e* (–)	*w*_opt_ (%)	*ρ*_d max_ (g/cm^3^)	*e* (–)	
grSa	9.00	2.020	0.31	8.50	2.085	0.27	2.65
grSa + 1.5%C	8.90	2.120	0.25	8.40	2.130	0.25	2.66
grSa + 3.0%C	8.80	2.140	0.24	8.30	2.150	0.24	2.66
grSa + 4.5%C	8.70	2.170	0.23	8.10	2.176	0.23	2.67
grSa + 6.0%C	8.50	2.182	0.23	7.90	2.190	0.22	2.68

C—cement addition.

**Table 2:** Testing sequences for base or subbase material after [6].

Sequence Number	Confining Pressure (kPa)	Max. Applied Axial Stress (kPa)	Cyclic Stress σ_*cyclic*_ (kPa)	Number of Load Applications
0	103.4	103.4	93.1	500–1000
1	20.7	20.7	18.6	100
2	20.7	41.4	37.3	100
3	20.7	62.1	55.9	100
4	34.5	34.5	31.0	100
5	34.5	68.9	62.0	100
6	34.5	103.4	93.1	100
7	68.9	68.9	62.0	100
8	68.9	137.9	124.1	100
9	68.9	206.8	186.1	100
10	103.4	68.9	62.0	100
11	103.4	103.4	93.1	100
12	103.4	206.8	186.1	100
13	137.9	103.4	93.1	100
14	137.9	137.9	124.1	100
15	137.9	275.8	248.2	100

## Data Availability

The data presented in this study are available on request from the corresponding author.
